# RTINet: A Lightweight and High-Performance Railway Turnout Identification Network Based on Semantic Segmentation

**DOI:** 10.3390/e26100878

**Published:** 2024-10-19

**Authors:** Dehua Wei, Wenjun Zhang, Haijun Li, Yuxing Jiang, Yong Xian, Jiangli Deng

**Affiliations:** 1 School of Traffic and Transportation, Lanzhou Jiaotong University, Lanzhou 730070, China; lihaijun@mail.lzjtu.cn (H.L.); jiangyx@mail.lzjtu.cn (Y.J.); xianyong@mail.lzjtu.cn (Y.X.); dengjl7723@gmail.com (J.D.); 2 Key Laboratory of Railway Industry on Plateau Railway Transportation Intelligent Management and Control, Lanzhou Jiaotong University, Lanzhou 730070, China; 3 School of Transportation and Logistics, Southwest Jiaotong University, Chengdu 611730, China; zwj726@my.swjtu.edu.cn

**Keywords:** railway turnout identification, semantic segmentation, Deeplabv3+, lightweight network, attention mechanism

## Abstract

To lighten the workload of train drivers and enhance railway transportation safety, a novel and intelligent method for railway turnout identification is investigated based on semantic segmentation. More specifically, a railway turnout scene perception (RTSP) dataset is constructed and annotated manually in this paper, wherein the innovative concept of side rails is introduced as part of the labeling process. After that, based on the work of Deeplabv3+, combined with a lightweight design and an attention mechanism, a railway turnout identification network (RTINet) is proposed. Firstly, in consideration of the need for rapid response in the deployment of the identification model on high-speed trains, this paper selects the MobileNetV2 network, renowned for its suitability for lightweight deployment, as the backbone of the RTINet model. Secondly, to reduce the computational load of the model while ensuring accuracy, depth-separable convolutions are employed to replace the standard convolutions within the network architecture. Thirdly, the bottleneck attention module (BAM) is integrated into the model to enhance position and feature information perception, bolster the robustness and quality of the segmentation masks generated, and ensure that the outcomes are characterized by precision and reliability. Finally, to address the issue of foreground and background imbalance in turnout recognition, the Dice loss function is incorporated into the network training procedure. Both the quantitative and qualitative experimental results demonstrate that the proposed method is feasible for railway turnout identification, and it outperformed the compared baseline models. In particular, the RTINet was able to achieve a remarkable mIoU of 85.94%, coupled with an inference speed of 78 fps on the customized dataset. Furthermore, the effectiveness of each optimized component of the proposed RTINet is verified by an additional ablation study.

## 1. Introduction

The rapid growth of rail transit systems, alongside the increasing operational mileage, has underscored the importance of transportation safety and the quality of operation and maintenance. In this context, the reliable operation of railway locomotives is crucial for ensuring the overall safety and efficiency of a railway network, as well as for protecting passenger safety [1]. In China, train drivers are critical for train safety, with their working conditions greatly affecting railway performance. The escalating speeds and density of trains have amplified the workload of train drivers, potentially leading to safety incidents attributed to human error, such as negligence and fatigue [2]. The automotive industry’s utilization of AI-based environmental awareness technology to assist drivers in safe vehicle operation serves as a blueprint for the rail sector [3]. This technology has also been effectively utilized in structural health monitoring and assessment of infrastructure, including bridges [4]. Implementing similar technology could reduce driver workload and promote autonomous train systems. Unlike automobiles, trains require monitoring of transportation equipment, particularly turnouts, which connect multiple tracks and direct train movement by shifting rail positions. These components are crucial for train safety [5] and have higher damage rates due to their complex structure and continuous train impacts [6]. High-speed trains, being fast and heavy, require equipment capable of identifying turnouts, which current conditions often fail to provide. Environmental perception technology, used to detect future turnouts, can reduce the workload of shunting locomotive drivers and improve shunting safety.

Environmental perception technology has advanced autonomous vehicles in the automotive industry [7,8]. It is primarily utilized for detecting foreign objects [9] and identifying railway defects [10] but used less for monitoring critical transport equipment like turnouts. In [11], traditional image recognition techniques were employed to differentiate turnouts from conventional straight rails for subsequent state monitoring of turnouts. In [12], AI technology was leveraged for condition monitoring and fault detection of railway turns but not for unmanned turnout recognition. In [13], a vision-based voting rate recognition algorithm was examined, using a custom dataset to achieve intelligent recognition of rail transit turnouts. Several experiments were conducted to analyze the impact of data enhancement and the image resolution and training size on a model’s performance. In [14], a deep learning-based environment perception method for track turnouts was proposed, optimizing the anchor box’s aspect ratio through transfer learning to enable the model to perform well with small datasets. Finally, the focal loss function was incorporated to address the issue of unbalanced datasets. In [15], a lightweight, dense, multi-scale fusion network is suggested, focusing on the railway environment and achieving railway scene classification.

It can be concluded that machine vision has emerged as pivotal technology in addressing the complex challenge of railway turnout recognition. Despite the advancements, several challenges persist. Firstly, optimizing human–computer interaction is crucial to convey railway line conditions to drivers efficiently after target identification, which is vital for effective driving. Secondly, the scarcity of turnout-related datasets poses a significant challenge [16], as current datasets focus more on rolling stock, signals, and stations, hindering research on turnout identification. Third, accuracy is paramount [17], as a complex railway environment can obscure the geometric features of turnouts. Improving recognition accuracy and reducing model misjudgments are central research foci. Lastly, the inference speed of a network model is critical [18], as swift identification of distant turnouts is necessary for providing timely track information and managing potential hazards like turnout failures. Deep learning-based image semantic segmentation has progressed significantly, with applications in various fields [19]. It offers sophisticated pixel-level analysis, revealing pixel features and leveraging contextual and semantic characteristics for comprehensive visual content understanding, which is beneficial for railway track line analysis and turnout recognition. Among this form of analysis, fully supervised learning-based methods [20], such as DeepLabv3+ [21], which builds on DeepLabv3 [22] and incorporates an encoder-decoder structure with the Xception network [23], are widely used for their preservation of fine-grained features and enhancement of contextual semantic comprehension.

Hence, we can borrow the successful recipes of the DeepLabv3+ network to design an identification model of railway turnouts. Nevertheless, there are still some problems which need to be solved. Initially, a railway turnout identification model is intended for deployment on high-speed trains to assist drivers in perceiving and making decisions about the track conditions ahead. In this context, real-time performance is a critical factor. However, the original DeepLabv3+ model, with its large parameter set and prolonged inference times, struggles to meet the demands for efficient deployment and real-time perception. Secondly, the operational environment in railway settings is inherently complex, with turnouts and tracks which are in close proximity and exhibit similar textural features. This presents a challenge for effective discrimination, particularly under the influence of variable weather conditions, lighting, and across different perception distances. Hence, there is an imperative need for more robust global and local feature perception capabilities. Moreover, due to interference from peripheral equipment and the sky background, there is an imbalance between the foreground and background. The cross-entropy loss function used in the original model is not sufficient to address this impact effectively. Thus, it becomes essential to consider a more comprehensive loss function to optimize the model’s training and learning process. Therefore, it is necessary to enhance the segmentation precision and augment the inference speed of the model. These advancements are essential to ensure the model’s efficacy in real-world applications, providing a robust solution for practical use cases.

**Figure 1:**
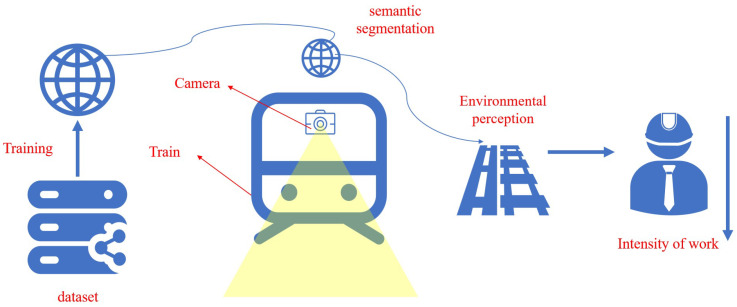
Research framework.

To solve these problems, a novel railway turnout identification network (referred to as RTINet) is proposed based on the improved end-to-end semantic segmentation model and prior knowledge, achieving real-time and high-precision identification of railway turnouts at the pixel level. At first, the construction of the railway turnout scene perception (RTSP) dataset commences with a polished selection from the RailSem19 [24] and GERALD [25] railway datasets, enriched by incorporating simulations of multiple weather scenarios. This dataset is then manually annotated and labeled, where the innovative inclusion of sidetrack recognition ensures a high standard of accuracy and consistency. Subsequently, the RTINet is presented, aligning with the refined architecture of the DeepLabv3+ network. It integrates a lightweight backbone network, depthwise separable convolutions [26], and bottleneck attention module (BAM) [27] and employs Dice loss [28] to enhance the original network with targeted improvements. These enhancements are designed to preserve the fine-grained features of segmentation and to better comprehend the contextual semantic information within images, resulting in excellent performance in terms of both segmentation accuracy and speed and thereby offering high practical application value. Finally, the effectiveness of the proposed RTINet is analyzed and demonstrated through multiple experiments. According to the experimental results, the RTINet demonstrates the capability to quickly and precisely identify railway turnouts at the pixel level, surpassing the performance of the benchmark models. This positions the RTINet as a highly viable alternative for practical applications. To summarize, the contributions and novelties of this paper are the following:

1. In tackling the challenge of turnout identification, this study adopts a human–computer interaction perspective. Deviating from conventional object detection methods, it employs semantic segmentation technology, which is more adeptly suited for driving scenarios. This approach yields feedback that is both more intuitive and comprehensive.

2. Given the scarcity of railway turnout datasets, a curated collection of 1000 turnout images is obtained through extensive screening of multiple railway datasets. To simulate various operational environments, a subset of these images is subjected to image processing simulations, replicating rain and fog conditions. This enriched dataset (named RTSP) is poised for application in turnout identification and status detection.

3. The manuscript introduces an innovative annotation methodology for turnouts, elucidating the significance of side rails. The model’s capability to discern side rails provides drivers with a clear perception of road conditions, thereby facilitating informed and accurate operational decisions.

4. Through an analysis of the key challenges in railway turnout recognition, this research presents a semantic segmentation-based railway turnout identification network (RTINet). This model is meticulously designed for expedited and precise identification of turnouts. It is intended to alleviate the workload of train drivers and enhance the safety of train operations, as graphically depicted in Figure 1.

The remainder of this paper is organized as follows. In Section 2, the main architecture of the proposed railway turnout identification network (RTINet) is presented first. Then, the RTINet is described in detail through an examination of four key components: the lightweight feature extraction network, the depth-separable convolutions module, the lightweight attention mechanism module, and the enhanced loss function. Each of these elements contributes to the model’s efficacy in achieving high-performance outcomes in the domain of turnout identification. In Section 3, the dataset, experimental environment, evaluation metrics, etc. as well as the model performance evaluation and comparison results are discussed. Finally, some conclusions for this paper are given in Section 4.

## 2. Methodology

**Figure 2:**
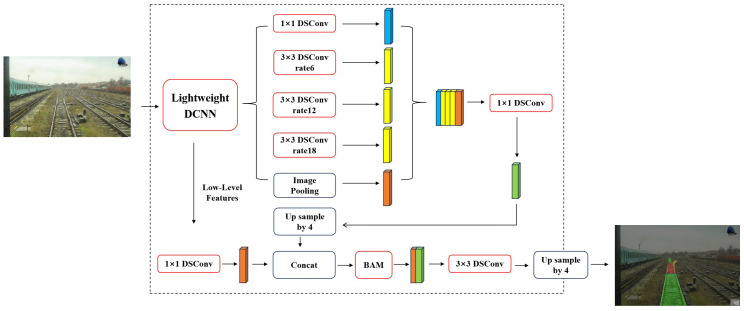
The main structure of the proposed RTINet model.

In response to the need for precise and effective railway turnout recognition, based on the Deeplabv3+ model, a railway turnout identification network (called RTINet) is proposed and evaluated in this paper. The primary framework of the proposed RTINet model is depicted in Figure 2. Concretely, the following targeted improvements have been made. The RTINet replaces the backbone network with the lightweight Mobilenetv2 and utilizes deep separable convolution to reduce computational costs. Furthermore, the model employs Dice loss as the loss function to address the imbalance between the foreground and background in turnout recognition without compromising the complexity of the model. The introduction of a lightweight attention mechanism (bottleneck attention module (BAM)) ensures that the model achieves high accuracy and a rapid detection speed. In this manner, the RTINet enhances the accuracy of railway turnout recognition, provides accurate railway line condition information, and significantly improves the model’s inference speed.

### 2.1. Lightweight Feature Extraction Network

**Figure 3:**
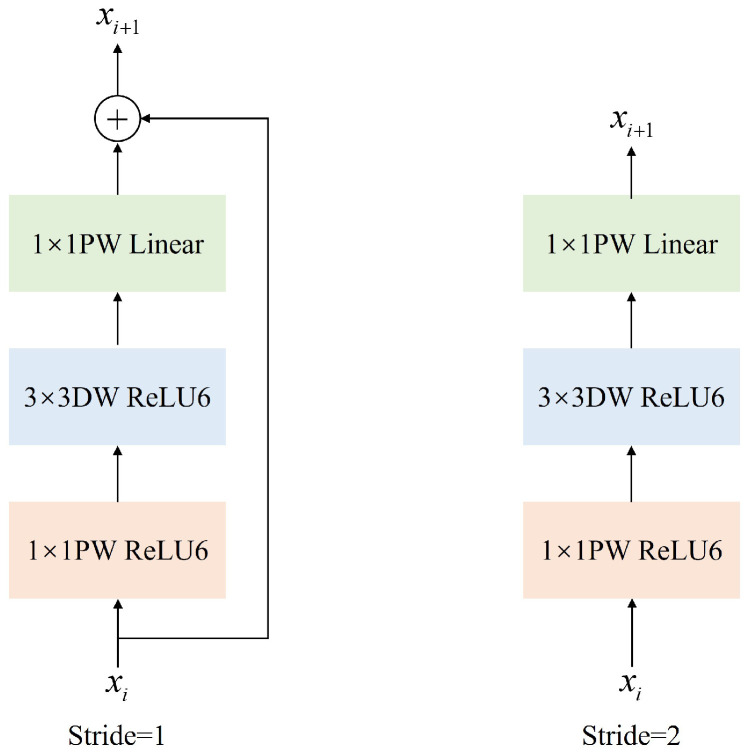
Structure of inverted residuals.

In light of the network model’s deployment on high-speed operating trains, this paper employed the MobileNetV2 network as the backbone network due to its optimality for lightweight deployment. Introduced by Google in 2018, MobileNetV2 [29] is an evolution of the classical neural network ResNet, incorporating an inverted residual structure as depicted in Figure 3. This structure typically involves reducing dimensions initially and then increasing them, with the convolution operation performed prior to image fusion to compensate for omitted information.

However, the application of residual structures within the context of deep separable convolutions can precipitate a notable loss of features. To counteract this, MobileNetV2 employs a 1 × 1 pointwise convolution at the outset to modulate the dimensionality of the feature map. This is succeeded by a depthwise separable convolution which extracts features. During the final phase of feature map generation, the feature map, which has been initially enhanced, is restored to its original channel count, culminating in the production of the final output. This methodical approach effectively curbs the gradient vanishing phenomenon within neural networks and bolsters the model’s capacity for feature extraction, all without a significant escalation in the number of network parameters.

The integration of MobileNetV2 as the backbone network thus confers dual advantages; it streamlines the network architecture, making it amenable for real-time processing on high-speed trains, while simultaneously preserving the integrity and richness of feature extraction, which is essential for accurate railway turnout detection and analysis. This strategic selection of the backbone network is pivotal for the model’s operational efficacy and its potential integration into autonomous railway systems.

### 2.2. Depth-Separable Convolutions

**Figure 4:**
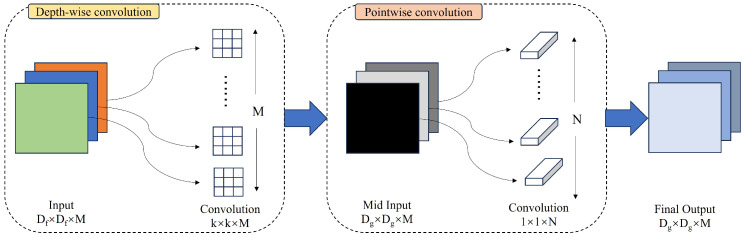
Depthwise separable convolution schematic diagram.

A conventional convolutional layer processes image data through the application of multiple multi-channel convolution kernels, yielding a feature map which encapsulates both channel and spatial features. In contrast, depthwise convolution operates on a per-channel basis, with the channel count remaining unchanged post processing for each input image, thereby facilitating the extraction of spatial information from the image data. Pointwise convolution, on the other hand, employs a 1 × 1 convolution kernel to process the image, preserving the image size while increasing the dimensionality of the feature map and channel features. The architectural framework of depthwise separable convolution is illustrated in Figure 4.

To describe in more detail, depthwise separable convolution amalgamates the principles of depthwise and pointwise convolutions. It employs depthwise convolution to process the input image and subsequently utilizes pointwise convolution to concatenate the channel features across all channels prior to output. A significant advantage of depthwise separable convolution over traditional convolution is its streamlined computational architecture, which is achieved by reducing the number of input channels. This reduction significantly decreases the parameter count required by the convolutional layer, leading to a more efficient model with a reduced parameter set and accelerated computational performance. These attributes render depthwise separable convolution highly adaptable for implementation across diverse computational platforms. Accordingly, in the RTINet architecture, depthwise separable convolution is utilized in place of standard convolution, thereby further enhancing the model’s inference speed. This strategic replacement leverages the efficiency of depthwise separable convolution to streamline the computational process, which is essential for real-time applications, where rapid analysis is critical.

### 2.3. Lightweight Attention Mechanism Module

**Figure 5:**
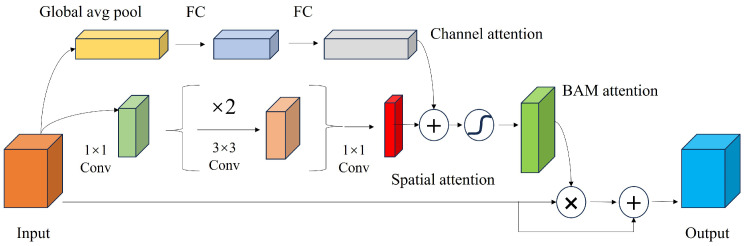
Network structure diagram of the bottleneck attention module (BAM).

The bottleneck attention module (BAM), as referenced in [27], is an enhancement module for deep neural networks designed to refine a feature map by augmenting spatial attention mechanisms and channel attention concentration. Its primary objective is to enhance the positional and feature information within a feature map. In the context of railway turnout recognition, it is essential for the network model to comprehensively understand the characteristics of the turnout and track and to accurately capture the position information of the locomotive’s path. Consequently, the integration of the BAM module, following the fusion of high-dimensional and low-dimensional features, effectively directs the network’s focus toward the salient features and their spatial context. This concept is graphically represented in Figure 5.

More precisely, the channel-wise weights within the BAM module serve to meticulously adjust the feature response of each channel, thereby amplifying pertinent information while concurrently suppressing noise. In parallel, spatial weights are engaged to modulate the feature responses across the spatial dimensions, enhancing the network’s sensitivity to critical regions within the imagery. The multiplicative combination of these weights is then applied to the original feature map, culminating in an output feature map which is significantly enhanced. This process is crucial for enhancing the network’s ability to discern and emphasize relevant features within a complex railway environment.

### 2.4. Loss Function

In the training of neural network models, the loss value functions as a quantitative metric to assess the discrepancy between the model’s predictions and the ground truth labels. A reduced discrepancy signifies more effective learning by the model. The model’s predictive output is generated through the forward propagation process, and the loss value, which quantifies the divergence from the actual label, is calculated using a loss function. This value is subsequently employed in the backpropagation process for updating the model parameters. In the realm of semantic segmentation, commonly employed loss functions include cross-entropy and the mean squared error. Specifically, cross-entropy is utilized in the Deeplabv3+ architecture to evaluate the divergence between labels and predictions, with the calculation formula defined as follows [30]:

(1) $$L_{CE}=\frac{1}{N}\sum_{i=1}^{N}-\left[y\log_{2}y^{\prime }+\left(1-y\right)\log_{2}\left(1-y^{\prime }\right)\right]$$

Nevertheless, traditional loss calculation methods are susceptible to the influence of sample imbalances, which can adversely affect the model’s training efficacy. In the operational context of a shunting locomotive, the focal area is confined to the space immediately ahead of the train, with the background comprising the surrounding tracks, equipment, and sky. A complex railway environment often results in a foreground-background imbalance, where the target area is relatively small. Employing a conventional loss calculation method may lead to minimal training errors but can result in suboptimal recognition performance in practical scenarios. To counteract this issue, a loss metric is introduced to address the challenge of data sample imbalance. More concretely, the Dice loss [28] is a metric derived from the Dice coefficient, which is utilized to quantify the similarity between two samples. A higher Dice coefficient value indicates a higher degree of similarity. The Dice coefficient is calculated using the following formula:

(2) $$Dice=\frac{2\left|X\cap Y\right|}{\left|X\right|+\left|Y\right|}$$

where *X* and *Y* represent the ground truth and the predicted situation, respectively; |X| and |Y| stand for the number of pixels identifying the target in a specific image for the ground truth and prediction, respectively; and $\left|X\cap Y\right|$ denotes the number of foreground pixels where *X* and *Y* intersect. Correspondingly, the Dice loss expression is defined as

(3) $$L_{Dice}=1-Dice$$

Overall, the model’s comprehensive loss function is formulated as the aggregate of the cross-entropy loss and Dice loss, effectively combining two distinct yet complementary metrics to optimize model training. This composite approach ensures a balanced emphasis on both the accuracy of classification and the spatial overlap between the predicted and true segmentation masks. The final loss function is thus expressed as

(4) $$Loss=L_{CE}+L_{Dice}$$

## 3. Experiments and Results

### 3.1. Introduction to RTSP Dataset

#### 3.1.1. Dataset Construction

At present, the availability of public railway datasets is constrained, with the majority concentrating on rolling stock, signals, and stations. There is a dearth of sample data pertaining to turnouts, and the compilation of railway datasets typically occurs during operational hours, which can result in a high degree of similarity among image data and introduce potential biases during a model’s training and validation phases. In this research, the RailSem19 [24] dataset and GERALD [25] dataset were strategically selected. Images of turnouts from the RailSem19 dataset were curated to align with the domestic railway operational context, while the intricate environments from the GERALD dataset were incorporated to ensure diversity. To bolster the model’s capacity to extract features from turnouts, the selection of track data was biased toward a higher proportion of curved tracks. This process culminated in the selection of 900 turnouts images and 100 track images for analytical purposes.

**Figure 6:**
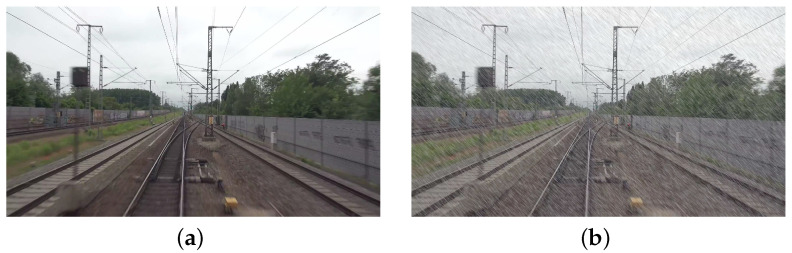
Schematic representation of the rainy day simulation effect. (**a**) Original image. (**b**) Simulated rainy day image.

**Figure 7:**
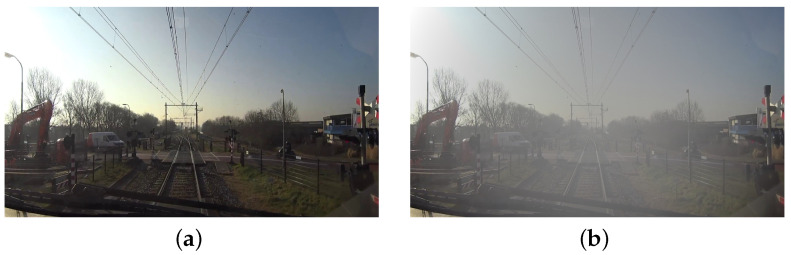
Schematic representation of the foggy day simulation effect. (**a**) Original image. (**b**) Simulated foggy day image.

Subsequently, given that trains operate under a variety of weather conditions, mainly including rain and fog, as well as under fluctuating lighting conditions, this study employs data augmentation techniques to simulate these environmental challenges. This approach is designed to enhance the model’s robustness in discerning turnouts and tracks under varied lighting scenarios. Data augmentation techniques included increasing the prevalence of rainy and foggy conditions within the dataset. Rainy day simulations were achieved by generating stochastic noise to mimic raindrops of varying sizes and orientations, whereas foggy simulations adjusted the luminance and contrast of pixels in a gradient from the image’s center outward to simulate a foggy atmosphere. To enrich the sample characteristics of the dataset, 100 models were randomly selected for foggy day simulation, 100 were selected for rainy day simulation, and 50 were selected for conditions encompassing both rain and fog. This comprehensive approach ensured that the trained model was equipped for effective performance in the round-the-clock operational railway environment. The simulation effects for rainy and foggy days are graphically represented in Figure 6 and Figure 7, respectively.

Ultimately, the aforementioned procedures culminated in the creation of the railway turnout scene perception (RTSP) dataset, comprising 1250 images. This dataset is not only reflective of real-world operational environments but also possesses the robustness necessary for the development and validation of sophisticated railway turnout recognition models. The RTSP dataset serves as a pivotal resource for advancing the state of the art in railway monitoring systems, ensuring that the models trained on this data are well equipped to handle the complexities and variations encountered in actual railway operations.

#### 3.1.2. Data Annotation

Nowadays, the literature on turnout annotation is characterized by a paucity of clear methodologies. Prevailing approaches often exhibit ambiguity in delineating the boundaries between turnouts and tracks, marking the contours of these elements, and are associated with laborious workloads. Furthermore, the identification of turnouts presents a significant challenge, which can impede the practical utility of annotation outcomes in aiding train operations.

**Figure 8:**
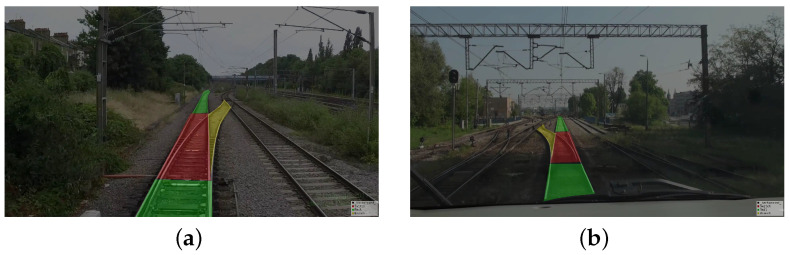
Example of labeling results with 3 categories. (**a**) Right side rail marking diagram. (**b**) Left side rail marking diagram.

Given all of this, an innovative turnout annotation methodology is introduced in this research. The defined scope of a turnout extends from the sharp rail to the termination of the turnout, with the novel concept of the side rail being employed to demarcate the region diverging from the current track. For operational purposes, train drivers must concentrate on the turnout and track sectors immediately ahead to maintain safety during navigation. Correspondingly, the predictive outputs of the network model are designed to emphasize the locomotive’s forward trajectory. In terms of data labeling, the focus is exclusively on annotating the turnouts, side rails, and tracks which lie ahead of the train. The enhanced annotation efficacy is depicted in Figure 8, where the green region indicates the track, the red region denotes the turnout, and the yellow region signifies the side rail. Table 1 provides a concise overview of the categorical distinctions within the turnout identification dataset.

### 3.2. Experiment Set-Up and Evaluation Metrics

The experimental framework was constructed utilizing the PyTorch deep learning library, which was accelerated by an NVIDIA GeForce RTX 3050 Laptop GPU [31] for model training. The input images were resized to 480 × 352 pixels, and the training was conducted with a batch size of four. Over the course of 200 epochs, the model was refined using the proposed comprehensive loss function in conjunction with the Adam [32] optimizer for the adjustment of network parameters. The learning rate was modulated using a cosine annealing decay strategy, initialized at a value of 5 $\times \;10^{-4}$, and scheduled to decrease to a minimum of 0.01 times the initial rate. The optimizer’s momentum was configured to 0.9, with the weight decay coefficient set to zero.

In the operational context of shunting locomotives, maintaining a panoramic view of the train’s immediate foreground is imperative, whether for assisting drivers in safe navigation or for the execution of unmanned operations. This includes the track, turnouts, and side rails. Autonomous detection of turnouts necessitates an ample braking distance to preemptively address potential safety hazards. Consequently, the rapidity with which the network model identifies turnouts is a paramount consideration. Therefore, this study concentrates on the evaluation of the model’s performance metrics, namely the mean intersection over union (mIoU), mean pixel accuracy (mPA), and frames per second (FPS), with particular emphasis on the intersection over union (IoU) and pixel accuracy (PA) across the three distinct categories. These metrics provide a comprehensive assessment of a model’s segmentation efficacy and its readiness for deployment in railway applications. Furthermore, this research employs precision and recall, which serve as critical metrics for evaluating the accuracy of a model’s pixel-level classification within images. These indicators furnish a comprehensive assessment of a model’s performance in accurately identifying and localizing each pixel to its corresponding class.

More specifically, the mean pixel accuracy (mPA) represents the average accuracy at the pixel level, which was obtained by calculating the pixel accuracy (PA) for each target and then averaging these values across the entire dataset. This metric provided a clear and direct reflection of the correctness of the prediction results. The calculation method is illustrated in the following formula:

(5) $$mPA=\frac{1}{k+1}\sum_{i=0}^{k}\frac{p_{ii}}{\sum_{j=0}^{k}p_{ij}}$$

where *k* denotes the number of target objects to be identified and $p_{ii}$ represents the count of pixels which are truly of class *i* and correctly predicted as class *i*, corresponding to the number of accurately detected pixels for that class. Conversely, $p_{ij}$ (where i≠j) signifies the count of pixels which are truly of class *i* but are incorrectly predicted as class *j*, thus indicating the number of pixels misclassified into a different category.

The mean intersection over union (mIoU) is a pivotal metric for assessing the performance of semantic segmentation tasks. It provides an intuitive evaluation of the segmentation outcome by calculating the ratio of the intersection to the union of the predicted and ground truth pixel regions for each image in the dataset. The higher the mIoU value, the greater the overlap between the predicted and actual regions, indicating superior segmentation accuracy. The computation of the mIoU is defined as follows:

(6) $$mIoU=\frac{1}{k+1}\sum_{i=0}^{k}\frac{p_{ii}}{\sum_{j=0}^{k}p_{ij}+\sum_{j=0}^{k}p_{ji}-p_{ii}}$$

Frames per second (FPS) is a metric utilized to evaluate the inference speed of semantic segmentation networks, representing the number of images which can be processed per second. Its calculation method is presented in Equation (7). The FPS metric is crucial for real-time applications, as it directly correlates with a model’s ability to swiftly analyze and respond to visual data. A higher FPS value indicates a more lightweight model, enabling faster and more timely turnout recognition:

(7) $$FPS=\frac{1}{T}$$

where *T* is the time in seconds needed to be perform inference on one image.

Within the context of semantic segmentation tasks, precision measures the accuracy of a model’s pixel predictions for a specific class; that is, it measures the proportion of pixels which truly belong to that class among all those predicted by the model to be of that class. Recall, on the other hand, assesses the comprehensiveness of a model’s detection for a particular class, namely the proportion of pixels correctly identified by the model out of all the pixels which actually belong to that class. Their calculation formulae are defined as follows:

(8) $$Precision=\frac{TP}{TP+FP}$$

(9) $$Recall=\frac{TP}{TP+FN}$$

where TP denotes the number of pixels correctly identified by the model as belonging to a specific class; FP indicates the number of pixels incorrectly labeled by the model as part of that class; and FN represents the number of pixels which belong to the class but were not detected by the model.

### 3.3. Performance Evaluation of the Proposed RTINet Model

**Figure 9:**
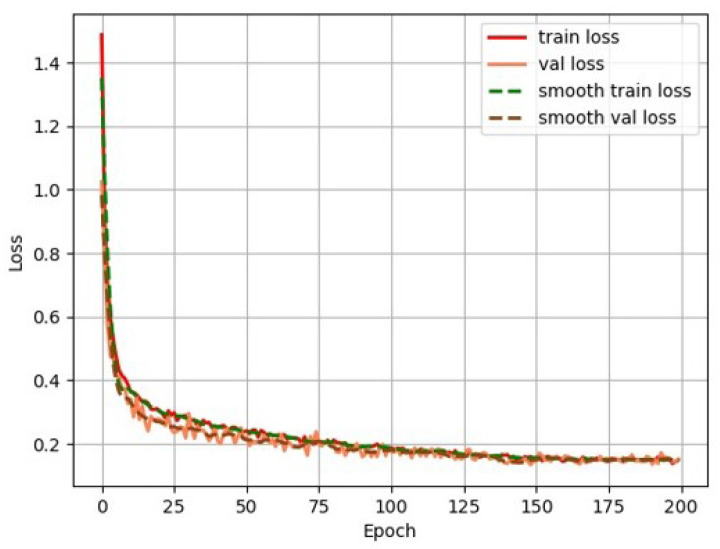
Loss curves of the RTINet model.

**Figure 10:**
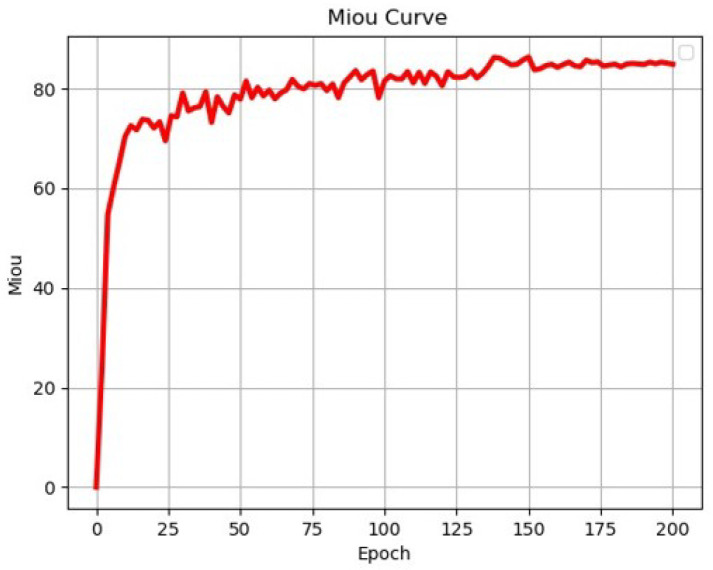
Validation mIoU of the proposed RTINet model.

To assess the effectiveness of the proposed method for railway turnout recognition, we compared it with the current mainstream semantic segmentation network model. For fair comparison, both models were trained using the RTSP dataset under the same environmental conditions. The comparison involved SegNet [33] using a convolutional encoder-decoder architecture, PSPNet [34] using ResNet50 [35] as the backbone network, Unet [36] using VGG [37] as the backbone network, HrNet [38] using parallel multi-resolution convolutions, and Deeplabv3+ using Xception [23] as the backbone network. The changes in each loss during RTINet model training are shown in Figure 9. Aside from this, the variation in the validation mIoU for the RTINet model during training is shown in Figure 10. Upon reviewing the values of the losses and the validation mIoU, it can be observed that the model achieved convergence after approximately 200 iterations. The stabilization of both curves confirms successful completion of the training process, indicating that the model learned the underlying patterns within the dataset effectively.

As delineated in Table 2, the RTINet model exhibited superior performance in terms of the intersection over union (IoU) values for the segmentation of tracks, turnouts, and side rails when juxtaposed with other state-of-the-art networks. Notably, the RTINet model achieved a 4% enhancement in its IoU for track segmentation, a 7% improvement in turnout segmentation, and a 6% increase in side rail segmentation relative to the DeepLabv3+ model. These gains underscore the RTINet model’s more equitable and robust recognition capabilities across all three critical components of railway infrastructure. Furthermore, the augmented IoU values signify that the RTINet model’s architecture and training regimen effectively addressed the nuances and complexities of railway turnout scene perception.

Table 3 illustrates a comparative analysis of the pixel accuracy (PA) for the segmentation of tracks, turnouts, and side rails among various network models. Each model exhibited a high degree of accuracy in its predictions. The RTINet model, as introduced in this study, significantly elevated the accuracy benchmarks established by the UNet architecture. The RTINet model’s enhancements yielded a notable increase in PA across all three categories: a 5% improvement for tracks, a 4% enhancement for turnouts, and an impressive 9% advancement for side rails. These improvements are not only substantial but also consistent, surpassing the accuracy levels achieved by other benchmark networks, including DeepLabv3+ with an Xception backbone. The advancements in PA provided by the RTINet model are a testament to its potential for improving safety and efficiency in railway operations.

Table 4 provides a comprehensive evaluation of the performance metrics for various models. The RTINet model demonstrated a marked enhancement over the already advanced DeepLabv3+ network model. Specifically, the RTINet model achieved an increase of 4.36% in the mean intersection over union (mIoU) and 3.17% in the mean pixel accuracy (mPA) relative to the DeepLabv3+ model. Furthermore, the RTINet model exhibited a substantial improvement in terms of frames per second (FPS), which is a critical parameter for real-time applications. The superior performance of the RTINet model across all three evaluation indices underscores its effectiveness in railway turnout scene perception. In particular, the significant boost in FPS suggests that the RTINet model not only provides high-quality segmentation but also does so with the speed necessary for real-time railway monitoring systems.

**Figure 11:**
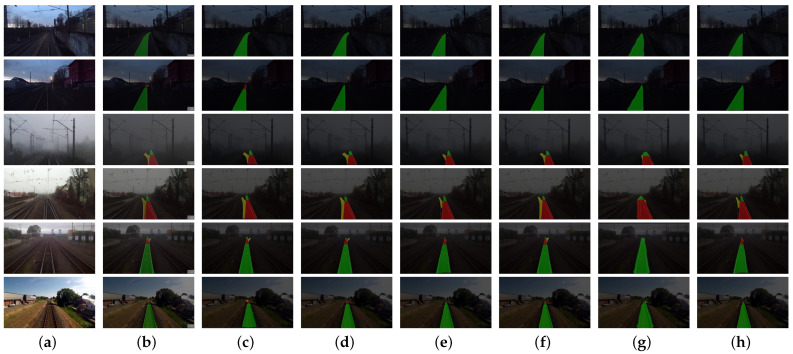
Qualitative comparison of segmentation results between baseline models and RTINet: (**a**) original; (**b**) ground truth; (**c**) RTINet; (**d**) Deeplabv3+ [21]; (**e**) HrNet [38]; (**f**) UNet [36]; (**g**) PSPNet [34]; and (**h**) SegNet [33].

For a comprehensive assessment of the enhanced network’s performance, a qualitative analysis methodology was applied to visually compare the segmentation results of the baseline networks against those of the RTINet model within the domain of turnout recognition. Figure 11 presents a comparative visual analysis, featuring six representative images which encapsulate a spectrum of conditions. This visual comparison serves to underscore the nuanced differences in the segmentation capabilities of the RTINet model relative to the established baselines, providing a qualitative benchmark for the model’s efficacy in diverse operational scenarios.

**Figure 12:**
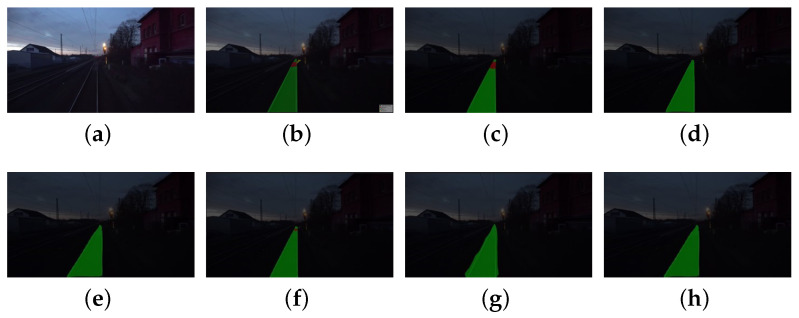
Comparison chart of segmentation results for tracks and turnouts under poor nighttime lighting conditions and at greater distances: (**a**) original; (**b**) ground truth; (**c**) RTINet; (**d**) Deeplabv3+ [21]; (**e**) HrNet [38]; (**f**) UNet [36]; (**g**) PSPNet [34]; and (**h**) SegNet [33].

To elaborate, the initial scenario featured less-than-ideal nighttime track illumination, where the RTINet showcased its capability by precisely identifying the track area even at significant distances. This precision is essential for timely detection and response mechanisms. In the second scenario, the challenge was posed from the perspective of a driver, where the visibility of an approaching turnout was diminished due to low-light conditions. The RTINet distinguished itself in this context, alongside the UNet architecture, as one of the few models capable of discerning distant turnouts. Nevertheless, as shown in Figure 12, UNet’s recognition range was found to be constrained, which could result in inadequate warning for drivers. The third scenario depicted a turnout under foggy conditions, a prevalent environmental factor known to obscure visibility. While several networks faltered in identifying the side rails, the RTINet sustained a higher degree of accuracy in regional classification, underscoring its resilience against inclement weather. The fourth scenario involved a shunting locomotive navigating toward a turnout. Here, the RTINet delivered more accurate predictions within the pivotal side rail area, ensuring precise path prediction and facilitating safer navigation. The final two datasets underscored the critical nature of long-range turnout identification to allow for sufficient braking distance post detection. The RTINet not only effectively recognized the turnout but also maintained clear recognition of the side rail area at greater distances, affirming its dependability and accuracy in railway surveillance systems. Collectively, the RTINet’s performance across these varied scenarios highlights its potential as an indispensable tool for bolstering railway safety and operational efficiency, especially under challenging environmental conditions.

In addition, the complexity of a model is an essential aspect to consider during evaluation, encompassing time and space complexity. Time complexity denotes the number of floating-point calculations, while space complexity refers to the number of model parameters. The CNN’s computational cost is defined as follows [39]:

(10) $$Time∼O(\sum_{l=1}^{D}M_{l}^{2}\cdot K_{l}^{2}\cdot C_{l-1}\cdot C_{l})$$

(11) $$Space∼O(\sum_{l=1}^{D}K_{l}^{2}\cdot C_{l-1}\cdot C_{l}+\sum_{l=1}^{D}M^{2}\cdot C_{l})$$

where $M^{2}$ represents the size of the feature map; *D* and *l* are the depth of model and the *l*th convolutional layer, respectively; *K* denotes the kernel length; and $C_{l}$ stands for the channel number of *l*th convolutional layer. Table 5 shows a complexity comparison of each model. The RTINet is distinguished by its rapid calculation speed, minimal parameters, and overall low complexity, making it well suited for deployment in locomotives to achieve turnout recognition.

### 3.4. Ablation Study

To substantiate the efficacy of the four enhanced methodologies presented in this manuscript, a series of ablation studies was conducted. The outcomes of these experiments are articulated in Table 6, which juxtaposes the performance metrics of the DeepLabv3 network with an Xception backbone against those achieved by the incremental enhancements. These enhancements included replacement of the backbone network, the adoption of depthwise separable convolutions, incorporation of Dice loss, and integration of the lightweight attention mechanism module (BAM).

Substitution of the backbone network precipitated amelioration across all three performance indices. Although the introduction of depthwise separable convolutions led to a modest decrement in the mIoU and mPA, it concurrently engendered a notable enhancement in the detection velocity. The judicious selection of a loss function tailored for turnout recognition enabled the model to achieve heightened accuracy without compromising the frames per second (FPS) metric. Ultimately, incorporation of the lightweight BAM module served to refine the model’s precision while incurring a minimal reduction in FPS, thereby harmonizing the competing demands of accuracy and detection speed.

Overall, these findings collectively demonstrate the synergistic impact of the proposed enhancements, underscoring the potential of the RTINet model to deliver a balanced performance profile, which is pivotal for real-time railway turnout recognition and analysis.

## 4. Conclusions

In this research, the issue of automatic railway turnout identification at the pixel level was examined for the first time. A railway turnout scene perception (RTSP) dataset was constructed, incorporating simulations of diverse weather conditions and pioneering the inclusion of sidetrack annotations. Concurrently, a railway turnout identification network (designated as the RTINet) was proposed, which is predicated on an enhanced end-to-end semantic segmentation model. The RTINet model incorporates several innovative refinements; it replaces the conventional backbone network with the lightweight MobileNetV2 and employs depthwise separable convolutions to diminish the computational overhead. The model also integrates Dice loss as the loss function, effectively managing the foreground-background imbalance in turnout recognition without sacrificing model complexity. Lastly, incorporation of a lightweight attention mechanism, specifically the bottleneck attention module (BAM), ensures that the model achieves high accuracy and turnout detection. The empirical results demonstrate that the RTINet model, operating at the pixel level, can rapidly and accurately identify railway turnouts, presenting a superior alternative for practical applications. More specifically, the RTINet achieved an impressive mIoU of 85.94% along with an inference speed of 78 fps on the customized dataset. This performance surpassed that of traditional methods, positioning the RTINet as a viable candidate for railway infrastructure analysis, where expeditious and precise identification is crucial for operational efficiency and safety. The model’s adept balance of accuracy and speed renders it a robust tool for aiding in the decision-making processes of train drivers and for potential integration into future autonomous railway systems.

In the future, the model’s long-distance turnout identification precision will be a primary focus for enhancement. The RTSP dataset will undergo further expansion, with the aim of bolstering all-weather detection capabilities for turnout status monitoring and unmanned locomotive operations. Field tests will be instrumental in refining and validating these improvements. Additionally, in line with advancements in inspection technologies, we will investigate more complex panoramic perception challenges related to railway lines, utilizing the state-of-the-art SAM and its variant models.

## Figures and Tables

**Table 1:** The number of different categories in the RTSP dataset.

Category	Number
Rail	1344
Turnout	964
Branch	962

**Table 2:** Comparison of IoU values between the proposed model and the baselines.

Method	IoU (%)		
	Rail	Turnout	Branch
SegNet [33]	67	74	50
PSPNet [34]	51	61	20
UNet-vgg [36]	74	77	60
HrNet [38]	71	76	48
DeepLabv3+-xception [21]	80	78	69
RTINet	**84**	**85**	**75**

Note: Bold text indicates the best result under the IoU metric for each category.

**Table 3:** Comparison of PA values between the proposed model and the baselines.

Method	PA (%)		
	Rail	Turnout	Branch
SegNet [33]	80	85	71
PSPNet [34]	78	85	29
UNet-vgg [36]	88	89	79
HrNet [38]	86	85	63
DeepLabv3+-xception [21]	88	85	87
RTINet	**93**	**93**	**88**

Note: Bold text indicates the best result under the PA metric for each category.

**Table 4:** Comparison of identification performance between the proposed model and the baselines.

Method	Performance Metrics				
	**mIoU (%)**	mPA (%)	mPrecision (%)	mRecall (%)	FPS
SegNet [33]	72.53	84.10	81.83	84.10	18
PSPNet [34]	57.82	72.55	67.54	72.55	36
UNet-vgg [36]	77.53	88.75	84.70	88.75	15
HrNet [38]	73.57	83.34	83.71	83.34	23
DeepLabv3+-xception [21]	81.58	90.07	89.07	90.07	23
RTINet	**85.94**	**93.24**	**90.74**	**93.24**	**78**

Note: mPrecision and mRecall represent the average precision and recall of all targets, respectively. Bold text indicates the best result for each metric.

**Table 5:** The computational costs of the experimental comparison models.

Method	Time Complexity (GFLOPs)	Space Complexity (M)
SegNet [33]	327.1	29.5
PSPNet [34]	118.4	46.7
UNet-vgg [36]	452.3	24.9
HrNet [38]	90.9	29.5
DeepLabv3+-xception [21]	166.9	54.7
RTINet	**11.8**	**2.7**

Note: Bold text indicates the best result for each metric.

**Table 6:** Ablation study of each component in our proposed method on RTSP dataset.

Baseline	MobileNetv2	DSConv	Dice Loss	BAM	mIoU (%)	mPA (%)	FPS
✓					81.58	90.07	23
✓	✓				84.11	92.28	63
✓	✓	✓			83.17	92.19	82
✓	✓	✓	✓		84.13	**93.28**	**82**
✓	✓	✓	✓	✓	**85.94**	93.24	78

Note: Bold text indicates the best result for each metric.

## Data Availability

The authors do not have permission to share data.
